# Epigenetic age predicts depressive symptoms during the COVID-19 pandemic in the Canadian Longitudinal Study on Aging: importance of biological sex

**DOI:** 10.18632/aging.206337

**Published:** 2025-11-18

**Authors:** Cindy K. Barha, Teresa Liu-Ambrose, Amy M. Inkster, Ryan S. Falck, Joel S. Burma, Susan Kirkland, Lauren E. Griffith, Mary Thompson, Nicole E. Basta, Jacqueline M. McMillan, Cynthia Balion, Christina Wolfson, Parminder Raina, on behalf of the Canadian Longitudinal Study on Aging (CLSA) Team

**Affiliations:** 1 Barha Brain Health Laboratory, Faculty of Kinesiology, University of Calgary, Alberta, Canada; 2 Hotchkiss Brain Institute, University of Calgary, Calgary, Alberta, Canada; 3 Aging, Mobility, and Cognitive Neuroscience Laboratory, Department of Physical Therapy, University of British Columbia, Vancouver, BC, Canada; 4 Djavad Mowafaghian Centre for Brain Health, Faculty of Medicine, University of British Columbia, Vancouver, BC, Canada; 5 Department of Medical Genetics, University of British Columbia, Vancouver, BC, Canada; 6 BC Children’s Hospital Research Institute, Vancouver, BC, Canada; 7 School of Biomedical Engineering, University of British Columbia, Vancouver, BC, Canada; 8 Department of Community Health and Epidemiology, Dalhousie University, Halifax, Nova Scotia, Canada; 9 Department of Health Research Methods, Evidence, and Impact, McMaster University, Hamilton, ON, Canada; 10 McMaster Institute for Research on Aging, McMaster University, Hamilton, ON, Canada; 11 Department of Statistics and Actuarial Science, University of Waterloo, Waterloo, ON, Canada; 12 Department of Epidemiology, Biostatistics and Occupational Health, McGill University, Montreal, Quebec, Canada; 13 Division of Geriatric Medicine, Cumming School of Medicine, University of Calgary, Calgary, Alberta, Canada; 14 Department of Pathology and Molecular Medicine, McMaster University, Hamilton, ON, Canada

**Keywords:** epigenetic clocks, COVID-19, depressive symptoms, sex differences, aging, CLSA

## Abstract

Epigenetic age is a biological metric of overall health and may predict mental health responses to unprecedented stressors. We sought to determine whether epigenetic age acceleration can predict older adults’ trajectory of depressive symptoms before and during the COVID-19 pandemic, and whether sex differences exist.

Data from baseline (2012-2015), first follow-up (2015-2018), and COVID-19 Baseline survey (April-May 2020) and COVID-19 Exit survey (September-December 2020) of the Canadian Longitudinal Study on Aging were used. Epigenetic age was measured at the study baseline, and depressive symptoms were assessed at each of the four time points using the 10-item Center for Epidemiological Studies Depression Scale (CESD-10). Sex-stratified mixed linear models examined the effect of epigenetic age (measured by DNAmAge and Hannum Age) on changes in CESD-10.

The mean participant chronological age at study entry was 63±10 years (46% female). Unexpectedly, younger epigenetic age predicted increases in depressive symptoms from first follow-up to COVID-19 Baseline survey (p’s < 0.05) in females only. Higher epigenetic age was not related to changes in CES-10 score during that time period (p’s > 0.05).

These findings suggest epigenetic age is a biological factor that can identify females at risk for greater negative effects of major life stressors on mental health.

## INTRODUCTION

The Coronavirus disease 2019 (COVID-19) pandemic has had a significant impact on the mental health of the global population [1]. Several studies have shown that increased exposure to stressors and experiences related to the pandemic (e.g., job loss, physical distancing and isolation, and caregiver burden) [2, 3] has led to increased prevalence of depressive symptoms and psychological distress in specific groups of the population [3–8]. While COVID-19-related social restrictions have eased back to pre-pandemic levels, it is nevertheless imperative to understand factors that can help identify those most at risk for the negative mental health impacts of pandemic restrictions. This knowledge will not only help develop effective interventions for protecting mental health during subsequent global pandemics but will also aid more general public mental health initiatives by helping identify subgroups of individuals who may be more affected by major stress exposures.

Sex differences exist in depression, with females experiencing a two-fold higher likelihood of being diagnosed and presenting with more severe symptoms than males [9, 10]. This sex difference extends to the effects of the COVID-19 pandemic on depressive symptoms, with females across the globe showing greater increases in depressive symptoms in several large cohort studies [4, 11–14]. While the etiology of this sex difference is not well understood, psychosocial, environmental, hormonal, and anatomical factors likely play roles [15]. Epigenetics, or covalent modifications to DNA without alterations to the underlying sequence, has emerged as a potential biological mechanism related to vulnerability or resilience to depression [16, 17], which likely contributes to differences in prevalence and incidence of depression between females and males [18].

Subtle variations in DNA methylation across the epigenome have been linked to numerous diseases and disorders [19]. Whereas chronological age represents the time elapsed since birth and increases uniformly across individuals, epigenetic age is a biomarker of biological aging derived from DNA methylation levels at a set of predefined genetic loci, influenced by genetic, environmental, and lifestyle factors [19]. Although first-generation epigenetic clocks were designed to predict chronological age, divergence between the two measures—manifesting as epigenetic age acceleration or deceleration—is a common phenomenon. Accelerated epigenetic age, defined as positive deviations from chronological age, is generally associated with adverse health outcomes, including cardiovascular and neurodegenerative diseases, as well as increased mortality [20]. In contrast, epigenetic age deceleration is a more complex phenomenon. While often linked to slower biological aging and better health, it has also been observed in individuals exposed to early-life neglect and war, supporting its role as a multifaceted and context-dependent biomarker [21–23]. Importantly, epigenetic age can be considered a surrogate measure of health, and some metrics have been found to correlate with the overall burden of disease [24].

Additionally, one’s physical and mental health history is strongly reflected in epigenetic age and epigenetic age acceleration [25]. For example, exposure to chronic stress and major depressive disorder in early and middle age are both associated with accelerated epigenetic aging in later life [26–28], and cumulative lifetime stress is more strongly associated with accelerated epigenetic age than current stress in a cohort of African American individuals [29]. While specific DNA methylation signatures have been shown to identify cases of major depressive disorder accurately and also predict which individuals will later develop depression [30], currently, it is unknown whether epigenetic age acceleration, which is derived from DNA methylation signatures, is itself predictive of future changes in depressive symptoms or in the ability to respond to stress.

Although it is well-appreciated that the COVID-19 pandemic was associated with high levels of individual stress exposure, it remains unknown whether an individual’s epigenetic age is predictive of their mental health response to COVID-19-related stressors and experiences. Thus, we build upon a previous report that used data from the Canadian Longitudinal Study of Aging (CLSA) platform and found that middle- and older-aged adults were twice as likely to develop depressive symptoms during the COVID-19 pandemic compared with pre-pandemic rates [4]. Older adults were disproportionately affected by COVID-19 and impacted by the public health measures imposed during the early stages of the pandemic, stemming from a combination of factors, including increased physical health risks to SARS-CoV-2 infection, social isolation due to stricter adherence to distancing guidelines, and heightened emotional stress [31–33]. Thus, we aimed to determine whether epigenetic age at a given time point could predict subsequent mental health status or change in mental health status following major stressors. Specifically, we sought to determine whether epigenetic age acceleration could predict the trajectory of depressive symptoms among middle-aged and older adults during the early part of the COVID-19 pandemic. These observations, along with the hypothesis that epigenetic age may predict subsequent mental health response to a major stressor, led us to ask whether epigenetic age acceleration can predict an individual’s trajectory of depressive symptoms during the COVID-19 pandemic. Given known sex differences in (i) epigenetic aging acceleration, with males having higher epigenetic age acceleration than women [34, 35], (ii) depression prevalence (higher in females) [36], and (iii) physiological response to stress in older age, with females showing greater cortisol release [37], we also conducted exploratory analyses to examine potential sex differences in the relationship between epigenetic age and the trajectory of depressive symptoms.

## RESULTS

### Participant characteristics

Participant characteristics at baseline are presented in Table 1. The demographic proportions of the final sample included in the current analyses were within 2% of those in the complete Comprehensive Cohort, and all participants with epigenetic data who also completed the COVID-19 Survey entry/exit portions (Table 1). Exceptions included sex, which was within 5% between samples, and PASE total scores, which were ~35 points higher for the final sample in the current analyses and for all participants with epigenetics data who completed the COVID-19 Survey, compared to the total Comprehensive sample (Table 1). For the final sample in the current analyses, the mean chronological age was 63 ± 10 years, with a female representation of 46%. More than 80% of participants had a university degree or higher, and participants reported living with an average of 7 chronic conditions (range: 2-16). The mean baseline CESD-10 score was 4.4 ± 5.2, DNAmAge was 57.5 ± 8.7 and Hannum Age was 63.0 ± 12.1. Chronological age was strongly correlated with both DNAmAge (r=0.86) and Hannum Age (r=0.85). DNAmAge and Hannum age were correlated (r=0.87).

**Table 1:** Participant characteristics at baseline (2012-2015).

**Participant characteristic**	**All comprehensive cohort participants (N= 30,097)**	**COVID-19 survey participants with baseline epigenetic data (N= 879)**	**Final sample (N= 663)**
Mean Age (SD)	62.96 (10.25)	62.46 (9.66)	62.79 (9.84)
Males (n,%)	14777, 49.1%	430, 48.9%	358, 54.0%
Mean Body Mass Index (kg/m^2^) (SD)	28.06 (5.44)	28.64 (5.77)	28.49 (5.71)
Education (n,%)			
*Less than high school*	1643, 5.5%	30, 3.4%	22, 3.3%
*High school diploma*	2839, 9.4%	77, 8.8%	56, 8.4%
*Some university*	2238, 7.4%	74, 8.4%	52, 7.8%
*University degree or higher*	23327, 77.6%	698, 79.4%	533, 80.4%
Income Level (n,%)			
*<$20,000 per year*	4373, 15.3%	113, 13.4%	72, 11.3%
*$20,000-$50,000 per year*	10538, 36.9%	278, 33.0%	199, 31.3%
*$50,000-$100,000 per year*	9696, 33.9%	304, 36.0%	239, 37.6%
*$100,000-$150,000 per year*	2517, 8.8%	94, 11.1%	80, 12.6%
*>$150,000 per year*	1465, 5.1%	55, 6.5%	46, 7.2%
Mean Number of Chronic Conditions	7.36 (2.62)	7.31 (2.55)	7.04 (2.47)
Alcohol Intake			
*Non-Drinker*	3427, 11.7%	89, 10.3%	63, 9.7%
*Occasional Drinker*	3705, 12.6%	119, 13.8%	85, 13.1%
*Regular Drinker (at least once per month)*	22239, 75.7%	665, 75.9%	503, 77.3%
Smoking Status			
*Daily Smoker*	2088, 7.0%	58, 6.6%	35, 5.3%
*Occasional Smoker*	488, 1.6%	12,1.4%	11, 1.7%
*Former Smoker*	17900, 59.8%	530, 60.6%	397, 60.2%
*Never Smoked*	9445, 31.6%	275, 31.4%	217, 32.9%
Mean PASE Total Score^1^ (SD)	129.26 (131.20)	165.26 (79.65)	166.51 (81.84)
Mean CESD-10 Score (SD)	5.67 (7.64)	5.29 (5.63)	4.44 (5.24)
DNAmAge	-	57.18 (8.65)	57.49 (8.72)
Hannum Age	-	62.52 (11.98)	63.00 (12.07)

^1^Physical Activity Scale for the Elderly.

### Association of epigenetic age with later changes in depressive symptoms during the COVID-19 pandemic

The present analyses include CESD-10 data collected from participants at 1) CLSA baseline (2012-2015), 2) CLSA first follow-up (2015-2018), 3) COVID-19 Baseline survey (April-May 2020), and 4) COVID-19 Exit survey (Sept-Dec 2020). Epigenetic age was measured in samples collected at CLSA baseline (2012-2015).

**Figure 1:**
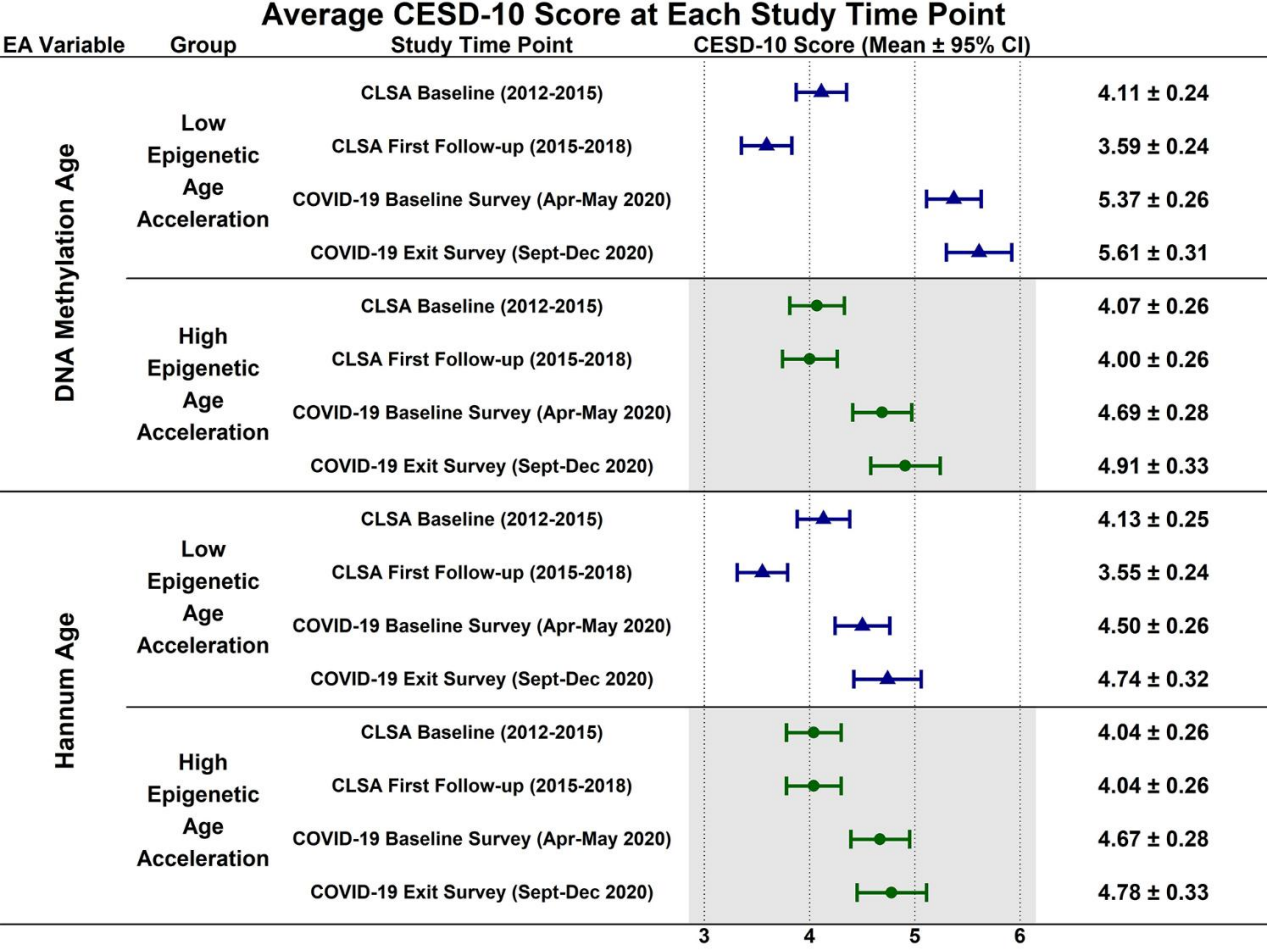
Estimated mean 10-item center for epidemiological studies depression scale (CESD-10) scores at each study timepoint for participants with low (-1 SD) and high (+1 SD) epigenetic age as determined by both epigenetic age markers.

**Figure 2:**
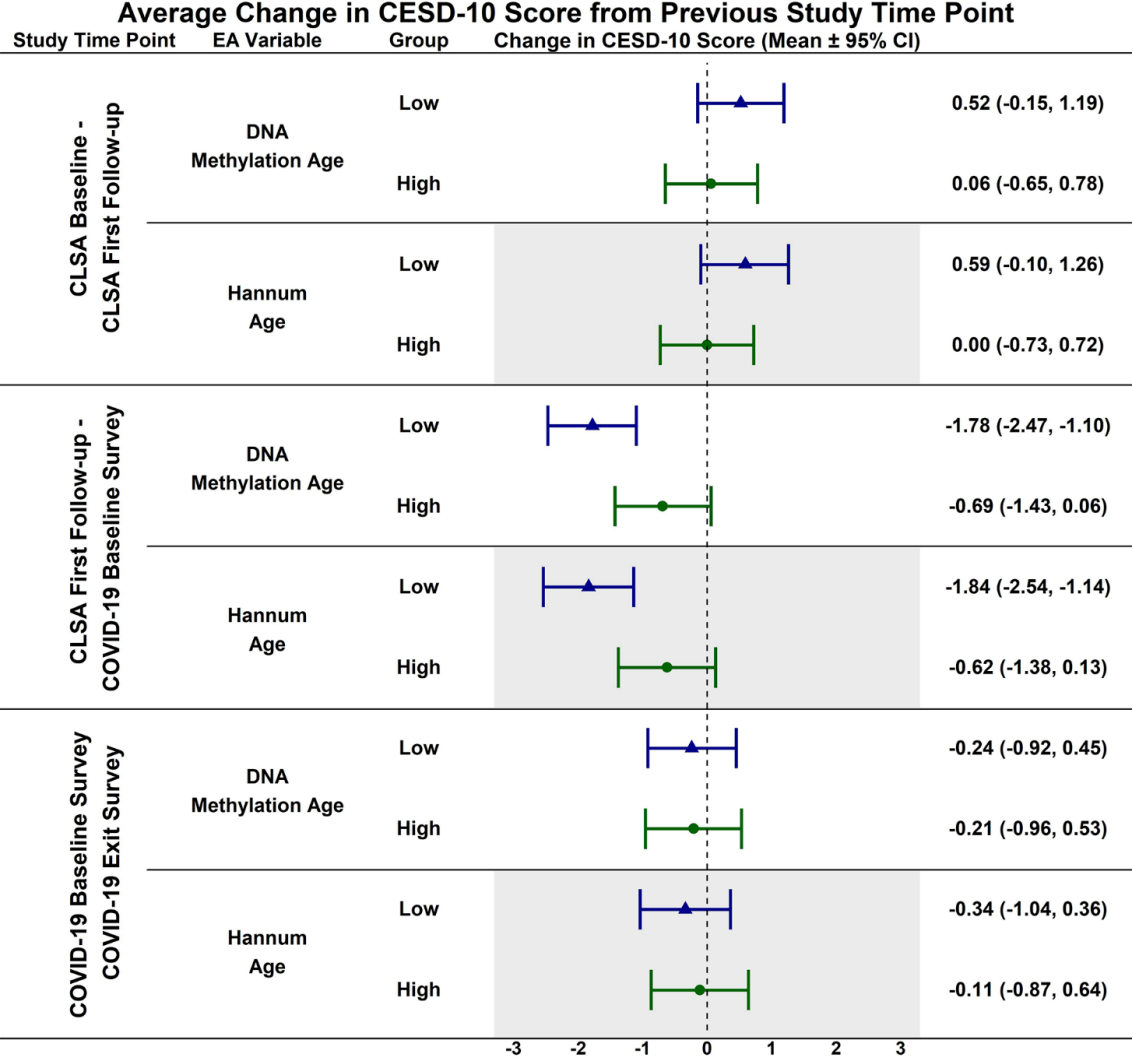
Estimated mean difference in 10-item center for epidemiological studies depression scale (CESD-10) score from CLSA baseline to CLSA first follow-up, from CLSA first follow-up to COVID-19 baseline survey, and from COVID-19 baseline survey to COVID-19 exist survey for low (-1 SD) vs. high (+1 SD) epigenetic age as determined by both epigenetic age markers.

Estimated CESD-10 scores for low and high epigenetic age at each time point are described in Figure 1; estimated changes in CESD-10 scores at each time point are described in Figure 2. CESD-10 scores did not change from CLSA baseline to CLSA first follow-up, from CLSA first follow-up to COVID-19 Baseline survey, or from COVID-19 Baseline survey to COVID-19 Exit survey for individuals with high epigenetic age, as determined by DNAmAge or Hannum Age.

In contrast, CESD-10 scores increased from CLSA first follow-up to COVID-19 Baseline survey for individuals with low DNAmAge (estimated mean difference: -1.78; 95% CI:[-2.47, -1.10], *p<0.001*) and low Hannum Age (estimated mean difference: -1.84; 95% CI:[-2.54, -1.14], *p<0.001*). Individuals with low DNAmAge (estimated mean difference: 1.10; 95% CI:[0.09, 2.11], *p<0.050*) and low Hannum Age (estimated mean difference: 1.22; 95% CI:[0.19, 2.25], *p<0.030*) had significantly greater increases from CLSA first follow-up to COVID-19 Baseline survey than individuals with high DNAmAge and high Hannum Age, respectively. CESD-10 scores did not significantly change from CLSA baseline to CLSA first follow-up or from COVID-19 Baseline survey to COVID-19 Exit survey for individuals with low epigenetic age, as determined by DNAmAge and Hannum Age.

### Sex differences in the association of epigenetic age with changes in depressive symptoms: exploratory analyses

**Figure 3:**
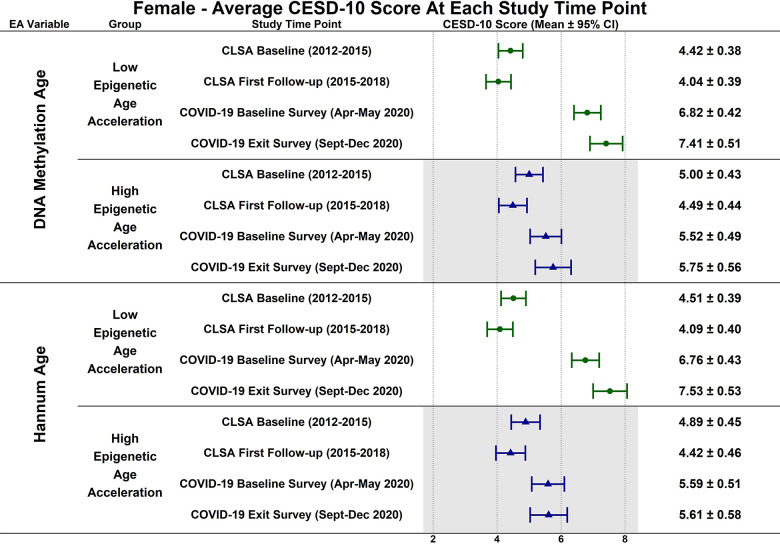
Differences in estimated mean 10-item center for epidemiological studies depression scale (CESD-10) scores in 305 females at each study timepoint for each epigenetic age marker based on low (-1 SD) and high (+1 SD) epigenetic age from exploratory, uncorrected analyses.

Estimated CESD-10 scores for low and high epigenetic age at each time point based on biological sex are described in Figures 3 (Female), 4 (Males); estimated changes in CESD-10 scores at each time point are described in Figures 5 (Females), 6 (Males). There were no changes in CESD-10 scores at each time point for males with high epigenetic age as determined by DNAmAge or Hannum Age. For males with low Hannum Age, CESD-10 scores increased from first follow-up to COVID-19 Baseline survey (estimated mean difference: -0.87; 95% CI:[-1.71, -0.03]). There were no significant differences in changes in CESD-10 scores between males with low and high epigenetic age categorizations.

**Figure 4:**
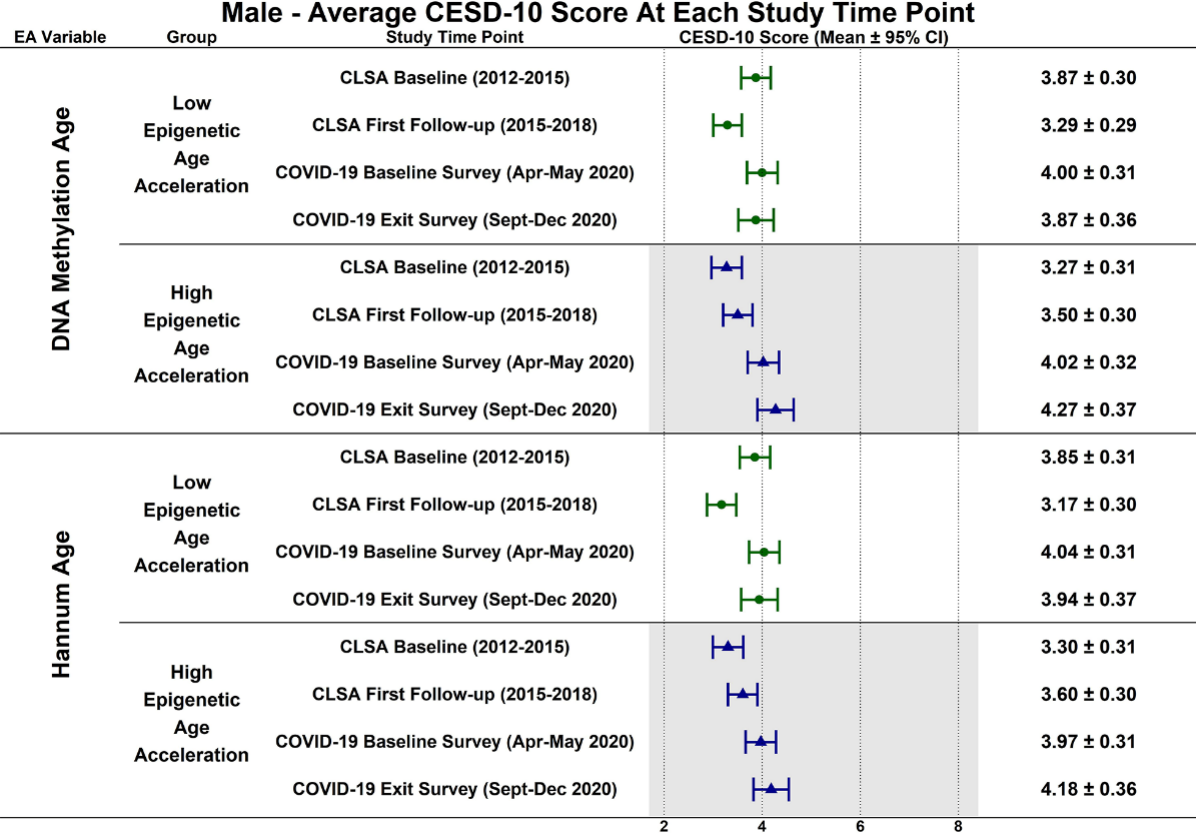
Differences in estimated mean 10-item center for epidemiological studies depression scale (CESD-10) scores in 358 males at each study timepoint for each epigenetic age marker based on low (-1 SD) and high (+1 SD) epigenetic age from exploratory, uncorrected analyses.

**Figure 5:**
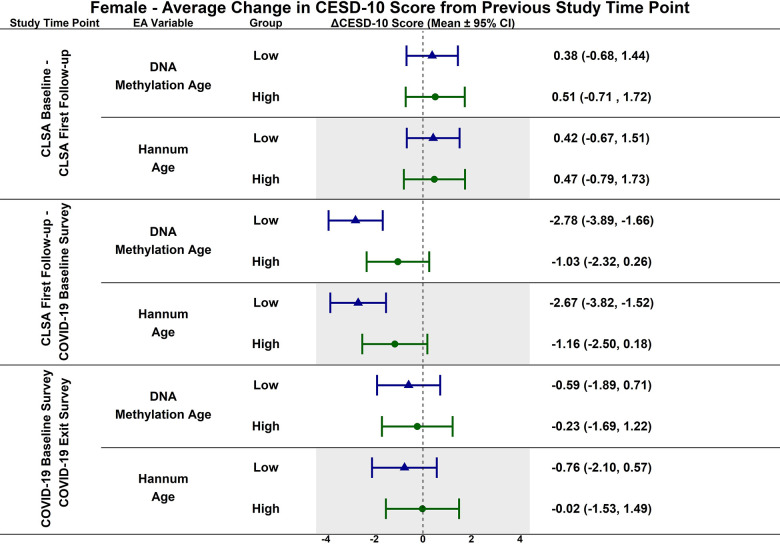
Differences in estimated mean difference in 10-item center for epidemiological studies depression scale (CESD-10) scores in females from CLSA baseline to CLSA first follow-up, from CLSA first follow-up to COVID-19 baseline survey, and from COVID-19 baseline survey to COVID-19 exist survey for low (-1 SD) vs. high (+1 SD) epigenetic age as determined by each epigenetic age marker from exploratory, uncorrected analyses.

**Figure 6:**
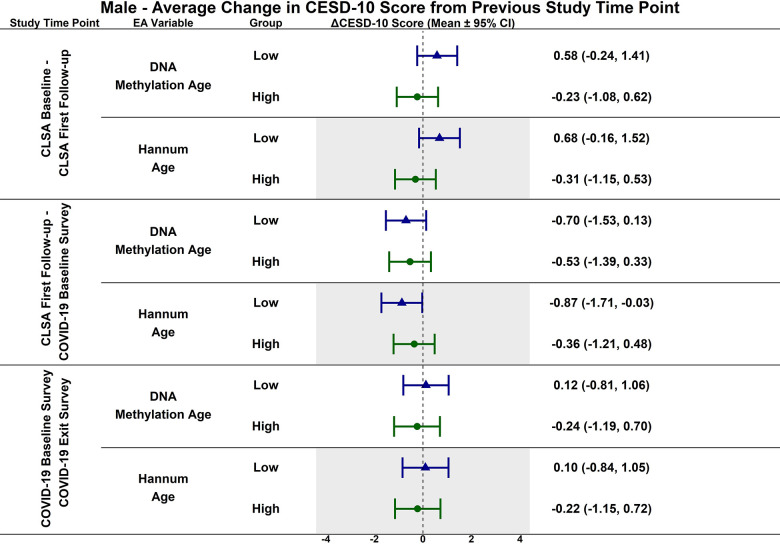
Differences in estimated mean difference in 10-item center for epidemiological studies depression scale (CESD-10) scores in males from CLSA baseline to CLSA first follow-up, from CLSA first follow-up to COVID-19 baseline survey, and from COVID-19 baseline survey to COVID-19 exist survey for low (-1 SD) vs. high (+1 SD) epigenetic age as determined by each epigenetic age marker from exploratory, uncorrected analyses.

CESD-10 scores did not change at any time point for females with high epigenetic age as determined by DNAmAge and Hannum Age. CESD-10 scores increased from CLSA first follow-up to COVID-19 Baseline survey for females with low DNAmAge (estimated mean difference: -2.78 95% CI:[-3.89, -1.66]) and low Hannum Age (estimated mean difference: -2.67; 95% CI:[-3.82, -1.52]). Females with low DNAmAge had significantly greater increases in CESD-10 scores from CLSA first follow-up to COVID-19 Baseline survey than females with high DNAmAge (estimated mean difference: 1.75; 95% CI:[0.04, 3.45]).

## DISCUSSION

The present study examined whether epigenetic age predicts an individual’s trajectory of change in mental health, specifically depressive symptoms, during the COVID-19 pandemic in people without a self-reported diagnosis of depression or anxiety at baseline, while considering biological sex as a modifying variable. For individuals with a younger epigenetic age, as estimated by DNAmAge and Hannum Age, there was a greater increase in depressive symptoms from the pre-pandemic period (i.e., 2015-2018) to the early stages of the COVID-19 pandemic (April–May 2020) compared to those with an older epigenetic age. Notably, the exploratory sex-stratified analysis demonstrated that the association between younger epigenetic age and increased depressive symptoms during the early stages of the COVID-19 pandemic was driven mainly by females who showed this relationship with both epigenetic age metrics. While extremely interesting, it is worth noting that these sex-stratified analyses are exploratory and have not been corrected for multiple comparisons; therefore, they should be viewed with caution.

Previous work in this CLSA cohort identified three distinct trajectories of change in depressive symptoms from pre-pandemic to the early-stages and later-stages of the pandemic, with some participants showing consistently low depressive symptoms, some showing moderate increases in depressive symptoms, and some showing large increases in depressive symptoms [4]. Importantly, compared with males, females were 2.17 times more likely to be in the group showing large increases in depressive symptoms and 1.70 times more likely to be in the group showing moderate increases than they were to be in the group showing consistently low depressive symptoms. The present analyses extend these findings by showing that in females, younger epigenetic age, measured several years earlier, predicted increases in depressive symptoms in response to the early stages of the COVID-19 pandemic. This suggests that in females, one’s cumulative history of physical and mental health as reflected in epigenetic age is a predictor of subsequent mental health, though the direction of the association was unexpected. Raina and colleagues found that males had lower odds of depressive symptoms during the pandemic [4], and here we found that male epigenetic age did not predict these changes in depressive symptoms.

In our analyses, older epigenetic age, as determined by DNAmAge and Hannum Age, did not predict changes in depressive symptoms during the COVID-19 pandemic. These results are contrary to our hypothesis, as older epigenetic age has previously been associated with negative mental health outcomes. For instance, adverse experiences and events in childhood are linked to increased depressive symptoms in older age, with accelerated epigenetic aging mediating this association [38]. Although it is important to note that this link is not always seen, as in some contexts, early life neglect and war exposure are associated with epigenetic deceleration [21–23]. One potential explanation for our findings could be the unique stressors of the pandemic, which may have influenced individuals differently than early-life adversities. Additionally, factors like social support, adaptive coping mechanisms, or increased resiliency due to previous adverse life events may have buffered the relationship between epigenetic aging and depressive symptoms during this period.

In contrast to the findings between high epigenetic age and depressive symptoms, Figures 1–6 highlight that younger epigenetic age predicts increases in depressive symptoms during the early stages of the pandemic. However, this association was only seen in females. Additionally, only in females was decelerated epigenetic aging associated with increased depressive symptoms in response to the COVID-19 pandemic. There are several possible reasons for this finding. First, factors that decelerate epigenetic aging may have amplified the negative effects of COVID-19 on mental health among females. For example, higher education and socioeconomic status are associated with younger epigenetic age as well as professional employment [39, 40]. Prior research shows that working outside the home prior to the pandemic is a risk factor for decreased mental health during the pandemic, especially for females [8]. This is likely due to the increased stress of balancing professional responsibilities with shifting work environments, while additionally contributing to childcare demands and family health concerns. The disruption of routines and adapting to additional pressures due to remote work may have contributed to heightened anxiety, stress, and depressive symptoms. As such, it is possible that the epigenetically younger females in our cohort were consequently more impacted by the pandemic from a mental health perspective.

We further propose that several social and lifestyle-related environmental exposures, previously associated with younger epigenetic age and decelerated biological aging [41], may have contributed to increased depressive symptomatology when significantly disrupted, as occurred during the COVID-19 pandemic. Social factors, such as frequent social contact, strong social support, and higher levels of socialization, are linked to a younger epigenetic age [42]. During the pandemic, individuals with more socially active lifestyles prior to lockdowns experienced greater increases in depressive symptoms due to enforced isolation [43]. Lifestyle behaviors associated with epigenetic aging show a similar pattern. Regular physical activity and exercise are consistently linked to slower epigenetic aging [44], while physical inactivity and sedentary behavior are associated with accelerated aging [45]. These behaviors also correlate with mental health: physical activity is protective against depression, particularly in females [46], whereas sedentary behavior is a known risk factor for depressive symptoms [47], with some evidence suggesting a stronger effect in females [48]. Previous work using the CLSA Covid-19 survey data indicates that pandemic-related restrictions led to reduced physical activity and increased sedentary time among older adults [49], both of which were associated with poorer mental health outcomes [50]. Taken together, these findings support the hypothesis that the disruption of social and behavioral exposures known to promote epigenetic deceleration and healthy aging may have exacerbated depressive symptoms during the COVID-19 pandemic.

The associations between epigenetic age acceleration and exposure to various adverse health and environmental factors, including obesity, low socioeconomic status, frailty, and diabetes, are well established in the literature [22]. However, it is important to note that while these correlations are frequently observed, they are not universally consistent across all studies or populations. Factors such as genetic variability, lifestyle, or resilience mechanisms may underlie why some individuals do not exhibit accelerated epigenetic aging despite facing significant adversity. For example, in a recent meta-analysis of studies examining factors associated with epigenetic aging, posttraumatic stress disorder was found to be associated with deceleration of epigenetic age, as measured by the Horvath clock, but not with other clocks (e.g., Hannum clock) [22]. Furthermore, epigenetic age deceleration assessed with the Horvath clock was also found in schizophrenia, while the PhenoAge and GrimAge clocks were positively associated with advanced epigenetic age [22]. Both posttraumatic stress disorder and schizophrenia are commonly treated through prescription medications, which could contribute to the divergent findings between epigenetic clocks [51]. Hence, in the present study, unexplored factors such as medication use, pre-existing mental health illness (diagnosed or that may have gone undiagnosed), and other cohort-specific factors at the time of epigenetic age measurement may be playing critical roles in the relationship between low epigenetic age and future increases in depressive symptoms in response to the COVID-19 pandemic.

Prior work has shown that lower income is strongly linked to higher rates of depression [52], which were exacerbated during the pandemic [53]. Financial strain can amplify daily stress, limit access to mental healthcare, and augment social and emotional challenges, which all contribute to greater depressive burden. Raina et al., found that during the initial COVID-19 lockdown and subsequent reopening, lower income was associated with greater increases in the odds of experiencing depressive symptoms [4]. Although income was included as a covariate in our analyses, we did not explore a potential interaction between income and epigenetic age. Future work with larger sample sizes should address this to help determine whether epigenetic age interacts with socioeconomic status to predict depressive response to major stressors. Furthermore, future investigations using epigenetic clocks and outcome measures that are likely to be influenced by anxiety, stress, and/or other mental health diagnoses would benefit from including interactive effects with income and biological sex where possible.

While the results of the present analysis reveal a unique pattern between epigenetic age, depressive symptoms during the early part of the COVID-19 pandemic, and female sex, they should be interpreted with caution, as the study has several important limitations. Epigenetic age was only measured once at baseline, 5 to 8 years before the COVID-19 pandemic. The long time interval between epigenetic age measurement and the onset of the pandemic could reduce the predictive power of epigenetic clock measures, as other environmental and lifestyle exposures experienced during this period could theoretically have had a greater impact on depressive trajectories in some individuals than the COVID-19 pandemic restrictions themselves. We also do not have access to information regarding depressive symptomology or specific exposures prior to the measurement of the epigenetic clocks in these individuals. It would be of interest to see whether the ability of epigenetic age to predict changes in depressive symptoms would differ if the measurement had occurred at the onset of the pandemic. Future work should measure epigenetic age closer to major stressor exposure. As epigenetic age was only measured once, we were also unable to examine whether changes in epigenetic age over time were associated with changes in depressive symptoms. Additionally, the type of observational data we had access to does not allow us to address and exclude reverse causality, in which the outcome variable influences the exposure rather than the other way around. To further understand the interaction between changes in CESD-10 scores and epigenetic age, our analyses extracted simple slopes for individuals with low epigenetic age, defined as 1 SD below the mean, and those with high epigenetic age, defined as 1 SD above the mean. As the low and high epigenetic groups are the extremes of the range and have no direct clinical relevance, our results are limited in their generalizability. Another limitation of using epigenetic clocks is that each clock has a degree of measurement error, typically ranging from approximately three to five years [52, 54, 55]. This measurement error likely arises from technical variability (e.g., differences in sample handling or array platform) and biological variation (e.g., inter-individual and inter-tissue variation in DNA methylation patterns arising from multiple sources) [56]. Nevertheless, this measurement error likely would have introduced a non-differential measurement bias, where the small differences did not introduce a systematic bias favoring one group (e.g., low vs. high epigenetic age) over another [57]. Thus, this likely would have had minimal influence on the predictive accuracy of the utilized clocks. Finally, epigenetic age was only measured in a subset of participants of the CLSA, and of these 1445 participants, 879 consented to participate in the COVID-19 Questionnaire Survey study. We further excluded 216 participants who self-reported diagnoses of dementia, anxiety, depression or mood disorder, reducing our sample size to 663 from 879, which does reduce the statistical power of our analyses. To balance this reduced statistical power, excluding these participants with mood or neurodegenerative disorders may have also theoretically reduced bias in our findings, as some studies suggest that those with existing mental health conditions were at greater risk for depression and anxiety during the pandemic [58–60]. Thus, future replication in a larger sample with more complete longitudinal data is required, which will allow for more sophisticated statistical modelling to examine both potential linear and nonlinear relationships.

## Conclusion

In summary, this study provides novel insights into the relationship between epigenetic age based on first-generation epigenetic clocks and depressive symptoms in response to the COVID-19 pandemic, particularly highlighting the potential sex-specific nature of these associations. The main findings were that younger epigenetic age (1 standard deviation below the mean), rather than older epigenetic aging, predicted increases in depressive symptoms during the early stages of the pandemic. Our exploratory analyses suggest this was only seen in females. These results underscore the importance of considering biological sex and its interaction with epigenetic markers in mental health research. Despite the study’s limitations, which include a single time-point measurement of epigenetic age 5-8 years before the COVID-19 pandemic and a relatively small sample size, both of which influence the interpretation of our findings, these results provide a foundation on which epigenetic markers can be used to identify individuals most vulnerable to the negative effects of major stressor exposures on mental health. We have proposed several hypotheses centred around the concepts of social epigenetics and the social exposome that could help explain our findings. However, these remain strictly hypothetical and require further study. Epigenetic clocks are promising biomarkers for inclusion in risk prediction for many different negative health outcomes and could also be utilized as a screening tool to determine the effectiveness of health-promoting interventions [61]. Specifically, within the context of mental health protection and promotion, epigenetic age based on first- and second-generation clocks, which allow for a more nuanced analysis, could be effectively deployed as a screen for at-risk groups, such as females with high occupational and caregiving burdens. Providing a better explanation for these associations will be crucial in informing public health strategies and developing targeted mental health interventions.

## MATERIALS AND METHODS

### Study design and participants

The CLSA is a longitudinal, national study of community-dwelling middle- and older-aged adults between the ages of 45-85 at baseline (2012-2015). Detailed study design and methods of the CLSA study have been described previously [62]. Very briefly, participants were recruited from across the 10 provinces through the Statistics Canada’s Canadian Community Health Survey, provincial health insurance registries, and random digit dialing. Exclusion criteria for recruitment in the CLSA were: 1) resident of the three territories of Canada; 2) unable to participate in English or French; 3) unable to provide their own data at baseline; 5) living on federal First Nations reserves or other First Nations settlements in the provinces; 4) full-time members of the Canadian Armed Forces; or 5) living in a long-term care institution [62, 63]. Of the 51,338 participants, a subset of 30,097 participants was part of the Comprehensive cohort and completed more detailed physical assessments and provided biospecimens at baseline (2012-2015). From the Comprehensive cohort, 1478 participants were randomly selected for DNA methylation analysis of blood samples provided at study baseline. The selected participants matched the distribution of the Comprehensive cohort in terms of province, age, and sex. Of the 1478 participants, the DNA methylation data from 1445 participants passed established quality control measurements. Of the 30,097 participants of the Comprehensive cohort, 27,765 provided data at first follow-up (2015-2018).

In response to the onset of the COVID-19 pandemic, the CLSA launched the COVID-19 Questionnaire Survey on April 15, 2020, to investigate the epidemiology of COVID-19 including its impact on the physical and mental health of older adults. Of the 51,338 participants recruited into the CLSA, 42,700 were viable at the time of the CLSA COVID-19 Questionnaire Survey and invited to participate. An additional 166 participants were found to be deceased, and 23 participants required a proxy to participate, thus they were excluded from participating. Therefore, 42,511 participants were eligible to participate in the CLSA COVID-19 Questionnaire Survey, of whom 28,559 completed the COVID-19 Baseline survey (April 15 – May 30, 2020), and 24,114 completed the COVID-19 Exit survey (September 29 - December 29, 2020). These data collected included information on COVID-19 symptoms and status, risk factors, healthcare use, health behaviours, and psychosocial and economic consequences of the pandemic. Of the CLSA participants who completed both the COVID-19 Baseline and Exit surveys, 18,533 were part of the Comprehensive cohort. We used data from the COVID-19 Baseline and Exit surveys.

**Figure 7:**
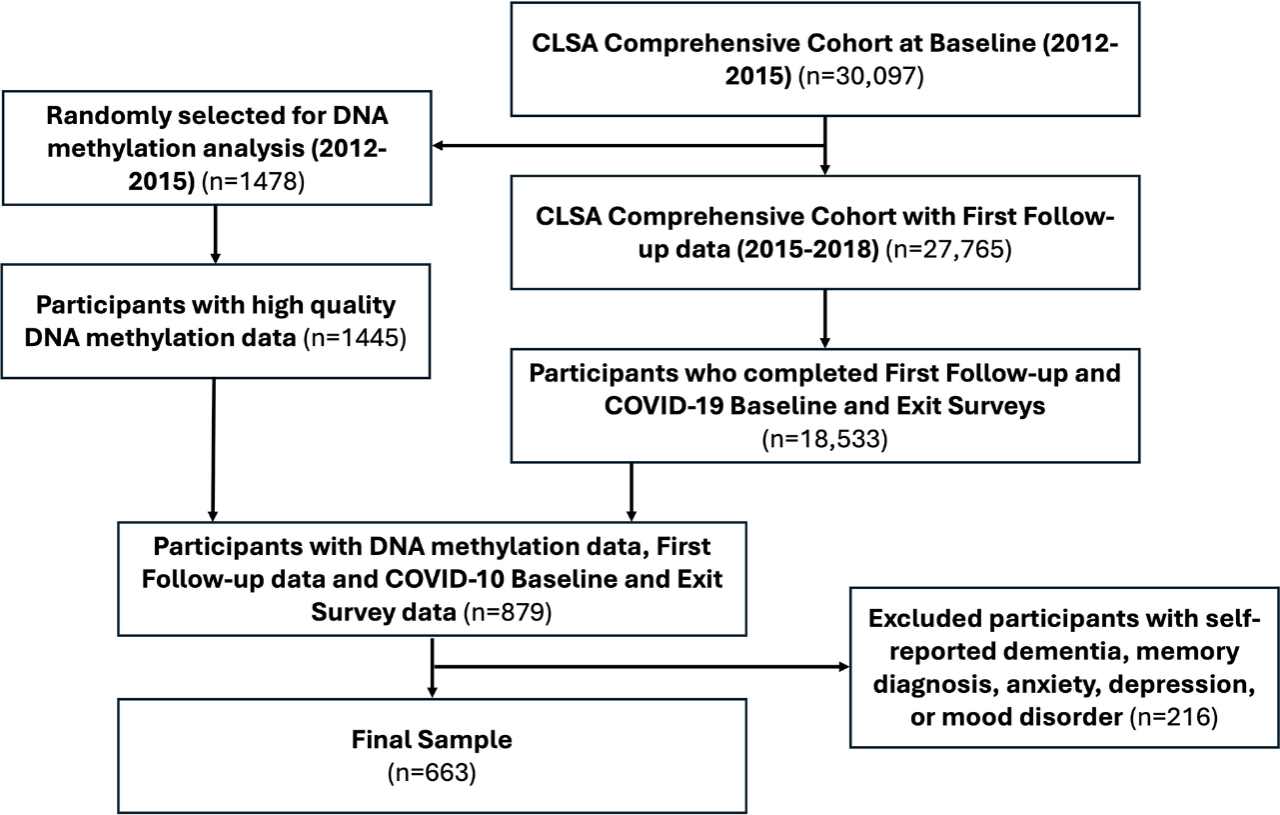
Strengthening the reporting of observational studies in epidemiology (STROBE) diagram.

Therefore, the present study includes participants from the Comprehensive Cohort (n= 30,097) that provided the required data for our analyses at 1) CLSA baseline (2012-2015), 2) CLSA first follow-up (2015-2018), 3) COVID-19 Baseline survey (April-May 2020), and 4) COVID-19 Exit survey (Sept-Dec 2020), as well as had DNA methylation data from blood at CLSA baseline (n= 879 out of the 1445 participants). In order to reduce reporting bias, participants who self-reported dementia or memory diagnosis or self-reported having been diagnosed with anxiety, depression, or mood disorder at study baseline (2012-2015) were excluded. Thus, the final sample size was 663 with the STROBE diagram presented in Figure 7.

### Measures

### DNA methylation and epigenetic age clocks

Blood samples from CLSA baseline (2012-2015) were assayed using the Illumina Infinium HumanMethylationEPIC (EPIC) BeadChip microarray (Illumina, San Diego, CA, USA), which profiles DNA methylation at >850,000 CpG sites genome-wide. A complete description of the procedures used to generate and prepare the DNA methylation data can be found here: https://www.clsa-elcv.ca/doc/3491. Raw DNA methylation data in the form of. IDAT files were downloaded and used to verify sample sex and identity. After using control probes on the EPIC array to confirm that sample sex matched reported sex, and that all samples were genetically distinct, we utilized epigenetic clock data derived by the CLSA team using weight and beta values that were normalized using the Noob normalization approach [64]. Specifically, for the present study we utilized Horvath’s DNA Methylation Age clock and Hannum’s epigenetic age clock [54, 55]. Briefly, Horvath’s DNA Methylation Age clock (DNAmAge) is a pan-tissue epigenetic clock based on DNA methylation at 353 CpGs, and was one of the first clocks to accurately predict chronological age of samples within 3.6 years, and has since its development been found to associate with biological age and is accelerated in association with many diseases and phenotypes including trisomy 21 [22, 65]. DNAmAGE is theoretically unconfounded with cell type proportions [55]. Hannum’s epigenetic clock (Hannum Age) is based on DNA methylation on 71 CpGs and was primarily developed for use with whole blood samples and has been associated with several complex diseases similar to Horvath’s clock [66]. For the present study, we used CLSA baseline (2012-2015) measurements of DNAmAge and Hannum Age.

### 10-item center for epidemiological studies depression scale

Depressive symptoms were indexed using the 10-item Center for Epidemiological Studies Depression Scale (CESD-10); a brief questionnaire with evidence of validity and reliability for measuring depressive symptoms [67, 68]. The CESD-10 is scored on a scale ranging from 0 to 30, with each of the 10 items rated on a 0-3 scale based on the frequency of depressive symptoms over the past week. Cut-offs typically include minimal depressive symptoms (0-9/30), mild depressive symptoms (10-15/30), and moderate-to-severe depressive symptoms (16-30/30) [69]. Participants completed the CESD-10 at the data collection site during the CLSA study baseline (2012-2015) and first follow-up (2015-2018). Additionally, participants completed the CESD-10 either by web or telephone during the CLSA COVID-19 Baseline survey (April-May 2020) and during the CLSA COVID-19 Exist survey (Sept-Dec 2020). For the present study, we used the continuous CESD-10 scores out of 30 from all 4 time points: CLSA study baseline, CLSA first follow-up, COVID-19 baseline, and COVID-19 exit.

### Covariates

At CLSA study baseline, participants’ age, biological sex, educational attainment (i.e., less than high school, high school diploma, some university, university degree or higher), income level, and current living status (i.e., house, apartment, assisted living, or other) were assessed. Additionally, at CLSA baseline participants were queried about whether they had ever been diagnosed with any of the following: heart disease, peripheral vascular disease, memory problems, dementia, multiple sclerosis, epilepsy, migraine headaches, intestinal or stomach ulcers, bowel disorders, bowel incontinence, urinary incontinence, macular degeneration, all-cause cancer, back problems which were not fibromyalgia or arthritis, kidney disease, arthritis in the hand, hip or knee, rheumatoid or other arthritis, any diabetes, high blood pressure, a thyroid condition, angina, stroke or transient ischemic attack, myocardial infarction, asthma, osteoporosis, parkinsonism or Parkinson’s disease, or chronic obstructive pulmonary disorder. The number of chronic conditions that a participant identified having been diagnosed with was then computed. In addition, body mass index (BMI: kg/m^2^) was measured using a calibrated scale and stadiometer. Participants were also queried at CLSA baseline for information on their smoking status (i.e., daily, occasional, former, or never-smoker), alcohol intake (regular, occasional, or non-drinker), and physical activity level as measured by the Physical Activity Scale for the Elderly (PASE) [70].

### Statistical analysis

All analyses were conducted in R version 4.0.3. Our primary analysis used the *lmer* package (version 1.1-25) and simple slopes analyses with the *Effects* package (version 4.2-2). Graphs and figures were plotted using the *ggplot2* package (version 3.3.5). We conducted all sensitivity analyses of epigenetic data using the packages *BiocManager* (version 1.30.16), *minfi* (version 1.36.0), *IlluminaHumanMethylationEPICmanifest* (version 0.3.0), *IllumniaHumanMethylationEPICanno.ilm10b4.hg19* (version 0.6.0), and *remotes* (version 2.4.1). Our complete statistical analysis and output can be found online (https://github.com/ryanfalck/CLSA-COVID-19-Epigenetics). We calculated means, standard deviations (SD), and proportions for all variables of interest at baseline.

We first examined changes in CESD-10 from CLSA baseline to COVID-19 Exit survey (i.e., four time points) based on epigenetic age using mixed linear models with restricted maximum likelihood estimation, wherein we included random intercepts and slopes. Time was also included as a categorical fixed effect. Separate models were conducted for each marker of epigenetic age (i.e., DNAmAge and Hannum Age). Each model controlled for biological sex, BMI, income level, educational attainment, living status, smoking status, alcohol intake, chronic conditions, and physical activity level. Significant time by epigenetic age interactions were then illustrated using simple slopes analyses in which the relationship of epigenetic age with CESD-10 score over time was estimated separately for low epigenetic age (i.e., 1 SD below the mean) and high epigenetic age (i.e., 1 SD above the mean) based on DNAmAge and Hannum Age separately. All estimates were calculated using the full model and included all covariates.

We then contrasted estimated changes in CESD-10 score from 1) CLSA baseline to CLSA first follow-up, 2) CLSA first follow-up to COVID-19 Baseline survey, and 3) COVID-19 Baseline survey to COVID-19 Exit survey for low versus high epigenetic age. We present contrasts as 95% confidence intervals. To account for multiple comparisons, the Benjamini-Hochberg procedure was applied to control the false discovery rate (FDR).

As an exploratory analysis, we examined whether biological sex moderated the relationship between epigenetic age and CESD-10 score over time. We conducted similar models while also including biological sex as an interaction term. We decomposed time by epigenetic age by sex effects using simple slopes analyses for low (-1 SD) and high (+1 SD) epigenetic age for males and females at each time point and contrasted estimated changes in CESD-10 score at each time point. As these were exploratory analyses, we did not control for multiple comparisons [71, 72].

### Sensitivity analyses

Sensitivity analyses were performed as quality control assessments of the EPIC data. Participant sex was assessed and confirmed to match annotated phenotypic data using fluorescence intensity values from microarray probes targeting the X and Y chromosomes, according to the method presented in the *ewastools* package [73]. Sample identity was assessed using the 59 explicit SNP genotyping probes included on the EPIC array to index contamination and sample mix-ups, this was also completed with *ewastools* functions [73]. EPIC array data indicated that there were no samples with contamination. We also determined that no samples had incorrect sex annotation. These results can be found on github https://github.com/ryanfalck/CLSA-COVID-19-Epigenetics).
